# Supplementary material for: Modeling glioblastoma heterogeneity as a dynamic network of cell states
Source: Mol Syst Biol. 2021 Sep 16;17(9):e10105. doi: 10.15252/msb.202010105 (PMC8444284; doi:10.15252/msb.202010105)
Supplement: Supplementary file 6 — Source Data for Figure 5 [file MSB-17-e10105-s004.zip › Figure5A_sourcedata/GSEA_3071/hallmarks_stateIII.GseaPreranked.1623742540963/HALLMARK_KRAS_SIGNALING_DN.html]

Details for gene set HALLMARK\_KRAS\_SIGNALING\_DN[GSEA]

|  || Dataset | state33071 |
| Phenotype | NoPhenotypeAvailable |
| Upregulated in class | na\_neg |
| GeneSet | HALLMARK\_KRAS\_SIGNALING\_DN |
| Enrichment Score (ES) | -0.4322484 |
| Normalized Enrichment Score (NES) | -2.0142715 |
| Nominal p-value | 0.0011350738 |
| FDR q-value | 0.0059383144 |
| FWER p-Value | 0.064 |
Table: GSEA Results Summary

  

Fig 1: Enrichment plot: HALLMARK\_KRAS\_SIGNALING\_DN      
 Profile of the Running ES Score & Positions of GeneSet Members on the Rank Ordered List

  

| PROBE | GENE SYMBOL | GENE\_TITLE | RANK IN GENE LIST | RANK METRIC SCORE | RUNNING ES | CORE ENRICHMENT || 1 | PDK2 |  |  | 491 | -0.254 | -0.1090 | No |
| 2 | PLAG1 |  |  | 623 | -0.261 | -0.1251 | No |
| 3 | SPHK2 |  |  | 660 | -0.263 | -0.1168 | No |
| 4 | NR6A1 |  |  | 683 | -0.264 | -0.1048 | No |
| 5 | ENTPD7 |  |  | 857 | -0.272 | -0.1310 | No |
| 6 | TAS2R4 |  |  | 1100 | -0.285 | -0.1741 | No |
| 7 | MSH5 |  |  | 1180 | -0.289 | -0.1750 | No |
| 8 | SNN |  |  | 1196 | -0.290 | -0.1595 | No |
| 9 | BTG2 |  |  | 1350 | -0.297 | -0.1789 | No |
| 10 | FGGY |  |  | 1406 | -0.300 | -0.1729 | No |
| 11 | TENM2 |  |  | 1734 | -0.321 | -0.2353 | No |
| 12 | LFNG |  |  | 1859 | -0.327 | -0.2452 | No |
| 13 | PTPRJ |  |  | 2589 | -0.381 | -0.4068 | Yes |
| 14 | CNTFR |  |  | 2640 | -0.385 | -0.3939 | Yes |
| 15 | DCC |  |  | 2702 | -0.391 | -0.3834 | Yes |
| 16 | YPEL1 |  |  | 2727 | -0.393 | -0.3632 | Yes |
| 17 | CLSTN3 |  |  | 2771 | -0.396 | -0.3478 | Yes |
| 18 | CCDC106 |  |  | 2925 | -0.412 | -0.3595 | Yes |
| 19 | SGK1 |  |  | 3150 | -0.437 | -0.3877 | Yes |
| 20 | EFHD1 |  |  | 3202 | -0.443 | -0.3712 | Yes |
| 21 | MFSD6 |  |  | 3316 | -0.464 | -0.3692 | Yes |
| 22 | ARPP21 |  |  | 3374 | -0.474 | -0.3521 | Yes |
| 23 | CELSR2 |  |  | 3378 | -0.475 | -0.3211 | Yes |
| 24 | MTHFR |  |  | 3509 | -0.511 | -0.3203 | Yes |
| 25 | SLC16A7 |  |  | 3635 | -0.568 | -0.3144 | Yes |
| 26 | TGFB2 |  |  | 3746 | -0.667 | -0.2980 | Yes |
| 27 | KLHDC8A |  |  | 3750 | -0.672 | -0.2538 | Yes |
| 28 | TFAP2B |  |  | 3792 | -0.729 | -0.2156 | Yes |
| 29 | GPRC5C |  |  | 3795 | -0.731 | -0.1672 | Yes |
| 30 | KCNQ2 |  |  | 3826 | -0.787 | -0.1223 | Yes |
| 31 | EDN1 |  |  | 3838 | -0.812 | -0.0708 | Yes |
| 32 | IGFBP2 |  |  | 3917 | -1.404 | 0.0031 | Yes |
Table: GSEA details [plain text format]

  

Fig 2: HALLMARK\_KRAS\_SIGNALING\_DN: Random ES distribution      
 Gene set null distribution of ES for **HALLMARK\_KRAS\_SIGNALING\_DN**
